# Natural Polyphenols From Moringa and Centella Extracts as Collagen Cross-Linkers and Matrix Metalloproteinase (MMP) Inhibitors in Dentin Preservation: An In Vitro Study

**DOI:** 10.7759/cureus.48530

**Published:** 2023-11-08

**Authors:** Lavanya A, Sindhu Ramesh, Venkata Suneel Kumar Kolaparthi, Muralikrishna Ch Nv, Shrikanya Rao KVL, Deepthi Mandava

**Affiliations:** 1 Conservative Dentistry and Endodontics, Narayana Dental College and Hospital, Nellore, IND; 2 Conservative Dentistry and Endodontics, Saveetha Dental College, Saveetha Institute of Medical and Technical Sciences (SIMATS) (Deemed to be University), Chennai, IND; 3 Oral Medicine and Radiology, Narayana Dental College and Hospital, Nellore, IND; 4 Conservative Dentistry and Endodontics, Lenora Institute of Dental Sciences, Rajahmundry, IND; 5 Biotechnology, College of Science and Technology, Vikrama Simhapuri University, Nellore, IND; 6 Conservative Dentistry and Endodontics, Faculty of Dentistry, Asian Institute of Medicine, Science and Technology (AIMST) University, Bedong, MYS

**Keywords:** enzyme-linked immunosorbent assay (elisa), collagenase, dentine collagen matrix, phenols, flavonoid, mmp inhibitor

## Abstract

Background

In adhesive dentistry, creating a long-lasting bond between resin composite and dentin is crucial. The durability of this bond dramatically depends on the structural integrity of collagen fibrils present in the hybrid layer. However, matrix metalloproteinases (MMPs) can degrade collagen fibrils, compromising the bond's longevity.

Aim

The objective is to evaluate the potential effectiveness of natural extracts from *Moringa* and *Centella* in preventing collagen degradation caused by MMPs.

Material and methods

The phenol and flavonoid content of the extracts were evaluated. Dentin beams were demineralized and pre-treated with 1% or 5% *Moringa*, 1% or 5% *Centella*, or 2% chlorhexidine (CHX) (five minutes), with untreated beams as control. Beams were incubated in calcium- and zinc-containing media (CM) at pH 7.2 and 37°C for one, 10, 20, and 30 days, and C-terminal cross-linked telopeptide of type I collagen (ICTP) release (collagen telopeptide) was assessed using an enzyme-linked immunosorbent assay (ELISA) kit after 30 days.

Results

Data were analyzed with a one-way analysis of variance (ANOVA). All test groups showed a different dry mass loss. The control group had the highest loss, followed by CHX, with the least loss in the 5% *Moringa* and *Centella* groups. ICTP release ranged from 1.781 ± 0.319 to 3.146 ± 0.684, with 5% *Moringa* showing the most negligible release.

Conclusion

The group that received 5% *Moringa* exhibited the most effective reduction in collagen degradation compared to all the other groups.

## Introduction

Successful bonding to dentin is a crucial aspect of modern adhesive dentistry, as it enables the formation of a hybrid layer through the complete infusion of resin monomers into water-saturated acid-etched dentin. The structural integrity and stability of collagen fibrils within the hybrid layer play a pivotal role in the durability of the resin dentin bond. However, the insufficient infiltration of collagen fibers by dental adhesive can expose collagen fibers, making them susceptible to collagenolytic activity by matrix metalloproteinases (MMPs). This can result in the degradation of the hybrid layer and ultimately deteriorate the bond strength [1].

The MMPs are the developmentally secreted inactive proenzymes (proteases) activated during the acid etching step in adhesive procedures [2]. During dentinogenesis, MMPs are responsible for the initial formation of dentin and the subsequent remodelling of the dentin matrix, playing a crucial role in tooth development. However, under pathological conditions, such as dental caries and periodontal disease, MMPs (2, 8, 9, and 13) can become overactive and contribute to the breakdown of the dentin matrix. Initial studies by Armstrong et al. utilizing transmission electron micrographs showcased the loss of insoluble collagen fibrils in the degrading hybrid layer [3]. The inhibition of the proteases or increasing the biochemical resistance of collagen structure is considered the primary approach for preventing the degradation of collagen fibers.

There has been extensive research on using collagen cross-linkers to improve the mechanical properties of demineralized dentin [4-6]. Cross-linking agents (e.g., proanthocyanidin, quercetin, and glutaraldehyde) are known to increase the stiffness of collagen by promoting additional hydrogen bonding and/or the formation of covalent inter- and intramolecular cross-links. Cross-linking prevents the long rodlike helical collagen molecules from sliding past each other under mechanical stress [7]. These agents have been found to improve the mechanical properties of demineralized dentin and have been proven to reduce biodegradation by endogenous proteases [5].

Plant extracts with polyphenols are being studied for their ability to stabilize collagen [8]. Polyphenols can help maintain the strength of resin dentin bonds and the integrity of demineralized collagen matrix, in addition to their antioxidant properties that inhibit MMPs [9]. Additionally, flavonoids (a type of polyphenol) can stabilize collagen chains and promote cross-links between collagen fibrils, which reduces collagen biodegradation [8]. Compared to synthetic, natural cross-linkers have gained favor due to their biocompatibility and absence of unwanted properties [8]. Glutaraldehyde is a well-known synthetic cross-linking agent for intermolecular covalent cross-linking, but concerns about cytotoxicity have limited its use [10].

Notably, polyphenols are effective MMP inhibitors by chelating zinc, which is essential for the functional activity of metalloproteinases [11]. The high biocompatibility and collagen cross-linking efficiency make polyphenols a favorable option for MMP inhibition. Additionally, plant flavonoids in whole plant extracts have been explored for topical use in inflammatory skin disorders for their MMP inhibition [12]. While previous studies have explored the use of collagen cross-linkers, the specific effect of natural polyphenols such as *Moringa oleifera* and *Centella asiatica* on MMP inhibition has not been explored.

Telopeptides are located at the ends of collagen molecules outside the triple helical structure of collagen fibrils [13]. They are made up of critical amino acids that help stabilize the collagen matrix and contribute to the mechanical properties of dentin. MMPs are responsible for breaking down collagen fibrils, creating telopeptides such as C-terminal cross-linked telopeptide of type I collagen (ICTP), and releasing them into the surrounding environment. Estimating telopeptide levels provides valuable insights into the relationship between telopeptides and collagen degradation [14]. The ICTP estimation method is an indirect quantitative measure of the functional activity of matrix-bound MMPs in situ, as opposed to western blotting and zymography [15].

The purpose of this research was to investigate how effective two natural polyphenols, *Moringa oleifera* and *Centella asiatica*, are at cross-linking and inhibiting MMPs. The study aimed to test the hypothesis that using these natural cross-linkers to pre-treat the demineralized dentin matrix would not inhibit MMPs, leading to the release of ICTP or reducing the loss of dry mass of the demineralized dentin matrix.

## Materials and methods

Plant collection and the preparation of plant extract

**Figure 1:**
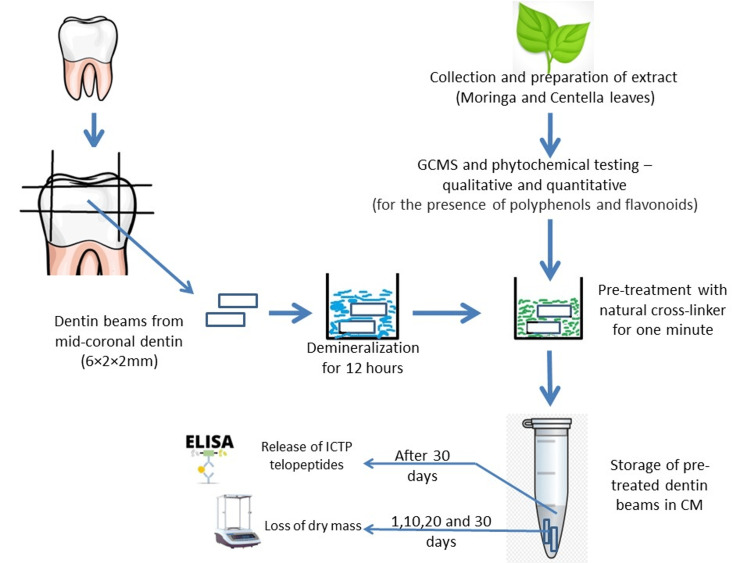
Graphical representation of the study. Image credit: Lavanya Anumula GCMS, gas chromatography-mass spectrometry; ELISA, enzyme-linked immunosorbent assay; ICTP, C-terminal cross-linked telopeptide of type I collagen; CM, calcium- and zinc-containing media

Fresh *Moringa oleifera* leaves were collected from a natural habitat near Nellore, while *Centella asiatica* leaves were obtained from the Indian Council of Agricultural Research (ICAR), Indian Institute of Horticultural Research, Bangalore. The leaves of both plants were destalked, shade-dried, and powdered using an electric blender. A Soxhlet apparatus was utilized to extract 100 g of each sample leaf powder with 100 mL of methanol. The extraction process lasted for eight hours until all soluble constituents dissolved in the solvent, indicated by the colorless siphon tube. Subsequently, the samples were concentrated using a rotary evaporator at 60°C until solvent recovery. The resulting sticky green extracts were stored in sterile containers at room temperature, for further use (Figure 1).

Gas chromatography-mass spectroscopy (GCMS)

The GCMS analysis of the methanolic extract of *Moringa oleifera* and *Centella asiatica* was performed using Clarus 500 (AutoSystem XL) (PerkinElmer, Waltham, MA) gas chromatograph coupled to a mass detector turbo mass gold (PerkinElmer's Turbomass 5.1 spectrometer with an Elite-1) (100% dimethylpolysiloxane). Samples were separated on a fused silica capillary column (30 m × 0.25 mm ID) with a film thickness of 1 µm. The column temperature was initially held at 110°C for one minute and then raised to 280°C, at a rate of 5°C/minute, maintaining it for nine minutes. The injector was set at a temperature of 250°C, and a split ratio of 10:1 was used with ultrahigh purity helium as the carrier gas at a flow rate of 1 mL/minute. The injection volume was 10 µL, and the mass spectral scan range was set at 45-450 (m/z) with an electron impact ionization voltage of 70 eV.

Phytochemical screening

Qualitative Screening

Phytochemical screening for phenols and flavonoids was performed on the extracted samples. Their quantitative estimation followed the confirmation of the presence of these phytochemicals.

Test for phenols: One percent ferric chloride was slowly added to the aqueous extract in a test tube and observed for color change. The development of a dark complex indicated the presence of phenols. The addition of ferric chloride to water, taken as a control, remained colorless.

Test for flavonoids: To the aqueous extract, 5 mL of dilute ammonia and 1 mL of concentrated sulfuric acid were added, and the solution was observed for the appearance of yellow coloration that disappeared on standing, indicating the presence of flavonoids.

Quantitative Evaluation

Estimation of total phenolics: The total phenolic content in the extracts was determined using the Folin-Ciocalteu procedure [16]. Samples (200 μL) were mixed with 1 mL of Folin-Ciocalteu reagent and 0.8 mL of sodium carbonate (7.5%), and absorption at 765 nm was measured. The total phenolic content was expressed as gallic acid equivalents (GAE) in micrograms per gram of extract.

Estimation of total flavonoids: The total flavonoid content was determined using a modified colorimetric method [17]. Test extract (1.0 mL) was mixed with 1 mL of distilled water and 75 μL of a 5% NaNO_2_ solution, followed by 75 μL of 10% AlCl_3_.H_2_O solution and 0.5 mL of 1 M sodium hydroxide, and absorbance was measured at 510 nm. The total flavonoid content was calculated using a standard quercetin calibration curve and expressed as micrograms of quercetin equivalents (QE) per gram of extract.

Dentin specimen preparation: Forty-five non-carious third molars were collected from individuals aged 18-45 with institutional review board approval (IEC/NDCH, 2019/P-24). The present study included multi-rooted extracted teeth with minimal coronal loss limited to enamel, devoid of cracks, while excluding teeth with structural anomalies and deformities. The selection criteria ensured that only teeth meeting the aforementioned parameters were included in the study. The teeth were cleaned, stored in distilled water with 0.02% NaN_3_ to prevent microbial growth, and used within two months of extraction. Dentin beams of 6 mm × 2 mm × 2 mm (length × width × height) were obtained from the mid-coronal dentin using a low-speed diamond cutter (model: Minitom, Struers, Copenhagen, Denmark) under water cooling. Each tooth yielded 1-2 beams of the required dimensions, resulting in 72 dentin beams. All dentin beams were completely demineralized in 10% phosphoric acid [18] for around 20 hours at room temperature, confirmed with a digital radiograph [19], and rinsed in distilled water for 20 minutes. To maintain consistent mean dry weights across all groups, the beams were randomly divided into six distinct groups, each with a sample size of n = 12. These groups were designated as follows: Group 1, consisting of 1% *Moringa oleifera*; Group 2, consisting of 5% *Moringa oleifera*; Group 3, consisting of 1% *Centella asiatica*; Group 4, consisting of 5% *Centella asiatica*; Group 5, consisting of 2% chlorhexidine (CHX); and Group 6, serving as the control group. The extracts were prepared to the required concentration using dimethyl sulfoxide (DMSO) (Figure 1).

The dentin beams in each group were treated with the respective test material for five minutes, except for the control group, where the beams were left untreated. Two percent chlorhexidine (CHX) was used as a positive control due to its known effectiveness against MMPs [20]. Following treatment, all the beams were blot-dried and placed in the labelled propylene tubes filled with 1 mL of calcium- and zinc-containing media (CM). These propylene tubes were placed in a shaking water bath and tested at 10-, 20-, 30-, and 45-day intervals. The CM composition was based on a previous study, which included 5 mM 4-(2-hydroxyethyl)-1-piperazineethanesulfonic acid (HEPES), 2.5 mM CaCl_2_.H_2_O, 0.02 mM ZnCl_2_, and 0.3 mM NaN_3_ (pH 7.2) [19].

Loss of dry mass: The loss of dry mass was used as an indirect method to measure the hydrolysis and solubilization of the dentin matrix after one, 10, 20, and 30 days of incubation [21]. After demineralization, the dentin beams were rinsed and dried, and the initial dry mass was measured using an analytical balance (ATX224 Uni Bloc, Shimadzu Corporation, Kyoto, Japan). Subsequently, the beams were rehydrated in distilled water, placed individually in labelled propylene tubes with CM, and incubated. After each incubation period (one, 10, 20, and 30 days), the beams were rinsed in distilled water to remove buffer salts and dried, and their dry mass was measured. This process was repeated after every incubation period, and the weight was expressed in grams up to four decimal points.

ICTP estimation: C-terminal cross-linked telopeptide of type I collagen (ICTP) was assessed to estimate matrix degradation by MMPs. An ICTP enzyme-linked immunosorbent assay (ELISA) kit from the Bioassay Technology Laboratory was used. This assay was based on sandwich enzyme-linked immunosorbent assay technology and was used to quantify solubilized type I collagen ICTP fragments released at the end of the 30-day ageing period. The release of telopeptide fragments from dentin over time has been established [22]. Therefore, the loss of dry mass was observed at regular intervals, and ICTP measurement was performed as a cumulative quantity when the loss of dry mass became insignificant after 30 days, i.e., when there is not much difference in 20- and 30-day interval dry mass loss. The ageing medium from the individual propylene tube was diluted 1:10 with saline and pipetted (50 μL per well) into a 96-well plate. Absorbance was measured at 450 nm using a plate reader (RT-2100 Microplate Reader, Rayto Life and Analytical Sciences Co., Ltd., Shenzhen, China). The standard curve was plotted using the kit's provided solutions and used to interpolate the target ICTP concentration of the samples, expressed as ng/mL.

Data analysis: The data collected from the experimental groups were subjected to statistical analysis using the Statistical Package for Social Sciences (SPSS) 20.0 software package (IBM SPSS Statistics, Armonk, NY) to evaluate the impact of natural extracts on dentin preservation and collagen degradation. A one-way analysis of variance (ANOVA) was employed to determine significant differences among the experimental groups, while post hoc multi-comparison analyses were conducted where appropriate. Box plots were generated to visually represent the data distribution and demonstrate variations in ICTP values across all experimental groups.

## Results

Plant extract

From 100 g of the initial powdered sample of *Moringa oleifera* and *Centella asiatica*, the yield percentages of the extracts were found to be 28% and 24%, respectively, using the Soxhlet apparatus. These extracts were later diluted to the required concentration using DMSO.

GCMS analysis

**Figure 2:**
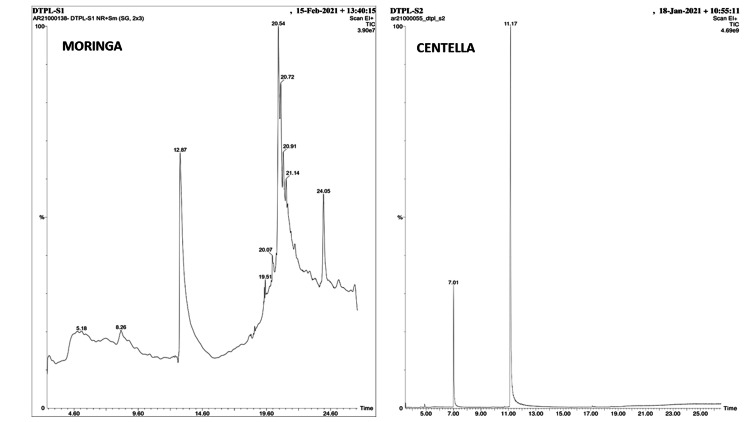
Chromatogram (GCMS) of Moringa and Centella. GCMS, gas chromatography-mass spectroscopy; DTPL, Dextrose Technologies Private Limited

The analysis identified and quantified the constituents of the extracts as a percentage of peak area and retention time. Figure 2 is the chromatogram showing 10 and two peaks in *Moringa* and *Centella*, respectively. These peaks were matched with standards available in mass spectrum libraries.

Phytochemical evaluation

Qualitative Analysis of Secondary Metabolites

Standard methods were used for the qualitative analysis of secondary metabolites in both *Moringa oleifera* and *Centella asiatica* extracts, revealing significant amounts of flavonoids and phenols.

Quantitative Analysis of Secondary Metabolites

The quantitative screening revealed a rich content of phenols (142.66 ± 7.80 mg and 137.66 ± 6.08 mg of gallic acid equivalents/gram of extract) and flavonoids (113.66 ± 5.50 mg and 103.66 ± 5.50 mg of quercetin equivalents/gram of extract), in *Moringa* and *Centella*, respectively. These identified secondary metabolites are known to possess antimicrobial, antidiabetic, and anticancer properties. Further analysis is needed to isolate, characterize, and elucidate the structure of bioactive compounds.

Loss of Dry Weight

**Figure 3:**
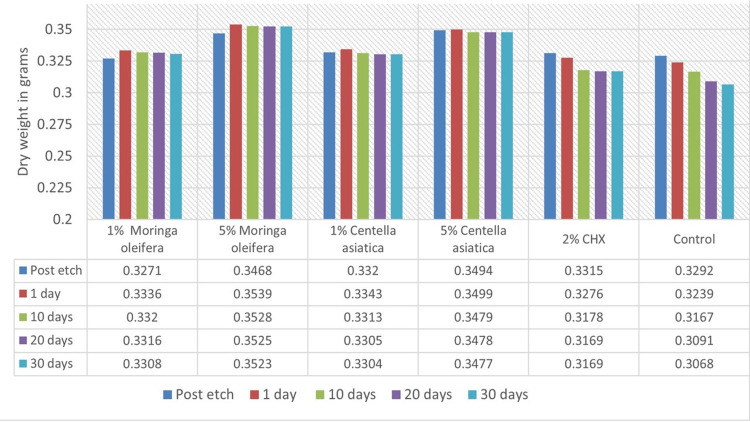
Loss of dry weight of dentin beams at various time intervals. CHX: chlorhexidine

The loss of dry mass served as an indicator to measure the protease activity. After soaking the samples in test solutions, an increase in dry mass was observed in the first four groups after a day, which was not observed in Groups 5 and 6. Subsequently, there was a gradual decrease in dry weight in all groups after 10- and 20-day intervals. The extracts with higher concentrations, viz., 5% *Moringa* (Group 2) and 5% *Centella* (Group 4), exhibited a smaller decrease in weight loss compared to the other groups. Groups 2 and 4 showed nearly stable dry weight between the 20-day and 30-day intervals. At the end of 30 days, the loss of dry weight (percentage) was recorded as follows: Group 1, 0.83%; Group 2, 0.45%; Group 3, 1.16%; Group 4, 0.6%; Group 5, 3.2%; and Group 6, 5.2% (Figure 3).

ICTP Estimation

**Figure 4:**
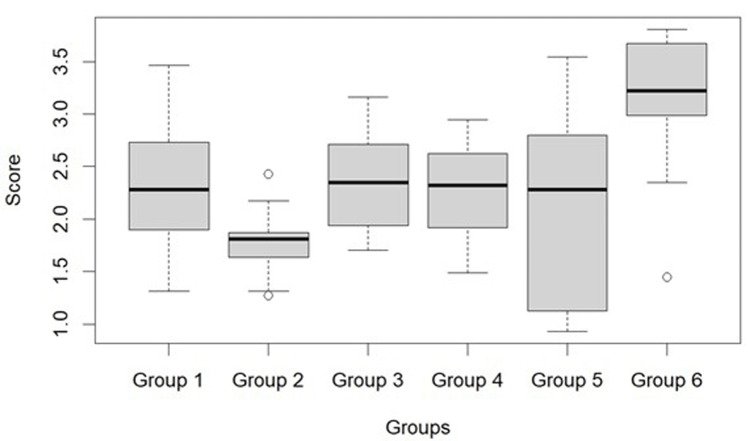
A box plot representing the ICTP release of all groups. ICTP: C-terminal cross-linked telopeptide of type I collagen

The ICTP estimation was performed after a 30-day interval. A one-way ANOVA analysis (Table 1) revealed a significant difference among all the groups (n ≤ 0.001). The mean liberation of ICTP in Group 6 (control) at the end of 30 days was 3.146 ± 0.684 ng/mg dry dentin, the highest value. The lowest value was observed in Group 2 (5% *Moringa*) (1.781 ± 0.319). Group 5 (CHX) also showed less ICTP release (2.056 ± 0.936). Post hoc multi-comparison analysis did not show any significant difference between Groups 2 and 5 (data not shown), but it did show a significant difference between Group 2 and Groups 1, 3, 4, and 6. Both concentrations of *Centella*, i.e., 1% and 5% groups, did not differ in activity and were similar to 1% *Moringa*. Figure 4 represents the box plot of the ICTP values of all the groups, showing the minimum value, maximum value, and second and third quartile, along with the median value of the released telopeptides.

**Table 1:** Mean of ICTP (telopeptides) released by all groups, as measured using ELISA and analyzed using one-way ANOVA. IQR, interquartile range; ICTP, C-terminal cross-linked telopeptide of type I collagen; ELISA, enzyme-linked immunosorbent assay; ANOVA, analysis of variance

	n	Mean (SD)	Median (IQR)	Minimum, maximum	P value
Group 1	12	2.335 (0.684)	2.281 (0.697)	1.316, 3.46	<0.001
Group 2	12	1.781 (0.319)	1.809 (0.225)	1.273, 2.431	
Group 3	12	2.353 (0.469)	2.345 (0.75)	1.702, 3.16	
Group 4	12	2.267 (0.49)	2.324 (0.59)	1.488, 2.946	
Group 5	12	2.056 (0.936)	2.281 (1.522)	0.93, 3.546	
Group 6	12	3.146 (0.684)	3.224 (0.622)	1.445, 3.803	

## Discussion

Polyphenols, occurring naturally in plants, have been extensively studied for their capacity to inhibit MMPs [23]. This study aimed to explore the collagen cross-linking and MMP inhibition properties of two natural extracts, *Moringa oleifera* and *Centella asiatica*. The results of this study demonstrated the efficiency of both natural extracts as collagen cross-linkers, leading to less mass loss and lower ICTP release. Notably, the 5% *Moringa* extract showed superior efficiency among the tested concentrations.

The GCMS analysis of the extracts revealed the presence of hydrocarbons, esters, alcohols, and ketones in *Moringa*, while *Centella* contained alkaloids, anthocyanins, and aromatic amides. Further phytochemical tests confirmed the presence of phenols and flavonoids known to possess MMP inhibitory properties. These phenols and flavonoids can increase the stiffness of dentin collagen fibrils, making them more resistant to unwinding, which is a desirable property for MMP inhibition [24]. The MMP inhibition and cross-linking properties of the extracts were assessed by measuring the loss of dry mass and solubilized telopeptide of collagen, respectively, following a previous study's approach [18].

The demineralized beams were treated with the respective cross linkers for five minutes for a more practical clinical application, unlike other studies where the dentin beams were treated for around 60 minutes [5,6]. The MMPs require calcium and zinc ions to maintain their tertiary structure and functional active sites [25]. The demineralized beams were thus placed in CM and maintained at pH 7.2 during the study period, for the enzymes to be active. To quantify the total degradation of the demineralized dentin matrix over time, the ICTP fragments that accumulated in the media were assayed at the end of 30 days. In their study, Garnero et al. observed that MMPs are the only source of ICTP telopeptide fragments from collagen matrices [13]. In this study, ELISA, an immunoassay test, was used to quantify the specific amount of degraded collagen peptides (ICTP), which works on the principle of antibodies to detect a specific protein in the sample.

It was observed that the pre-treatment with a natural cross-linker resulted in a slight increase in the dry weight after one day of storage in CM, unlike previous studies where a decline in the dry mass was observed in the first interval (Figure 3) [18,21,26]. This increase in dry weight could be attributed to the use of DMSO to dilute the extracts, causing the collagen matrix expansion [27]. Notably, the loss of dry mass during the storage period was not evident in the higher concentrations of the natural cross-linkers (5% *Moringa* and 5% *Centella*), and only a small decrease was observed in the lesser concentrations (1%). On the other hand, the control group (Group 6) and the group treated with CHX (Group 5) showed consistent loss of dry mass at different time intervals, with the CHX group exhibiting reduced loss compared to the control group.

The ICTP release analysis demonstrated that natural cross-linkers and CHX were effective in reducing telopeptide release compared to the non-treated (control) group. However, the relationship between the loss of dry mass and ICT release did not always correlate. For instance, Groups 2 and 4 exhibited the least dry mass loss, but only Group 2 (*Moringa*) showed the least telopeptide release. This suggests that while *Centella* (Group 4) is efficient as a cross-linker and effective against other proteases, it may not be as effective against MMPs, which are the primary cause of ICTP telopeptide release [28]. On the other hand, CHX, a nonspecific MMP inhibitor, exhibited a greater reduction in dry mass but similar ICTP release compared to Groups 1, 3, and 4. This suggests that CHX may not be an effective cross-linker but is a potent MMP inhibitor.

During the 30-day interval, some groups experienced a rise in the loss of dry mass. This might be because collagen degradation products diffuse out of demineralized dentin matrices, even after the proteases have been inactivated [29]. The rate at which collagen peptides dissolve decreases over time [30]. Therefore, a cumulative ICTP was evaluated at a 30-day interval.

The null hypothesis was rejected, as pre-treatment with natural cross-linkers effectively inhibited MMP activity, resulting in decreased ICTP release and less dry mass loss compared to the untreated group. Both *Moringa* and *Centella* extracts, rich in phenols and flavonoids, show potential as pre-treatment liners to minimize collagen degradation and enhance the durability of resin dentin bonds. The efficacy of these cross-linkers is concentration-dependent, with higher concentrations showing better inhibition of MMPs and reduced collagen degradation. *Moringa* extract at 5% concentration proved efficient in reducing mass loss and minimizing ICTP release.

One limitation of this study is the lack of assessment of cathepsin enzymes, which could be addressed in further research. These findings enhance our knowledge of natural extracts as potential cross-linkers, and further studies should correlate these cross-linkers with micro tensile bond strength tests to validate their effectiveness.

## Conclusions

The study concludes that natural cross-linkers, especially *Moringa* extract at 5% concentration, can effectively prevent MMP activity, leading to less dry mass loss and reduced ICTP release. This indicates that these extracts may improve the durability of resin dentin bonds by reducing collagen degradation. However, more research is needed to determine the effectiveness of these cross-linkers through micro tensile bond strength tests, followed by clinical trials.
